# The Influence of HLA Polymorphisms on the Severity of COVID-19 in the Romanian Population

**DOI:** 10.3390/ijms25021326

**Published:** 2024-01-22

**Authors:** Mihaela Laura Vică, Minodora Dobreanu, Ghenadie Curocichin, Horea Vladi Matei, Ștefana Bâlici, Mihaela Elvira Vușcan, Alin Dan Chiorean, Gheorghe Zsolt Nicula, Daniela Cristina Pavel Mironescu, Daniel Corneliu Leucuța, Cosmin Adrian Teodoru, Costel Vasile Siserman

**Affiliations:** 1 Department of Cell and Molecular Biology, “Iuliu Hațieganu” University of Medicine and Pharmacy, 400349 Cluj-Napoca, Romania; mvica@umfcluj.ro (M.L.V.); sbalici@umfcluj.ro (Ș.B.); mihaelacimpianu@yahoo.com (M.E.V.); Chiorean.Alin@umfcluj.ro (A.D.C.); gnicula@umfcluj.ro (G.Z.N.); dana_pvl@yahoo.com (D.C.P.M.); 2 Legal Medicine Institute, 400006 Cluj-Napoca, Romania; cvsiserman@gmail.com; 3 Emergency Clinical County Hospital, 540136 Târgu Mureș, Romania; minodora.dobreanu@umfst.ro; 4 Department of Laboratory Medicine, “George Emil Palade” University of Medicine, Pharmacy, Science and Technology, 540142 Târgu Mureș, Romania; 5 Center for Advanced Medical and Pharmaceutical Research, “George Emil Palade” University of Medicine, Pharmacy, Science and Technology, 540142 Târgu Mureș, Romania; 6 Department of Family Medicine, “Nicolae Testemițanu” State University of Medicine and Pharmacy, MD-2004 Chișinău, Moldova; curoc@usmf.md; 7 Emergency Clinical Hospital for Children, 400370 Cluj-Napoca, Romania; 8 Department of Medical Informatics and Biostatistics, “Iuliu Hațieganu” University of Medicine and Pharmacy, 400349 Cluj-Napoca, Romania; dleucuta@umfcluj.ro; 9 Clinical Surgical Department, Faculty of Medicine, “Lucian Blaga” University of Sibiu, 550169 Sibiu, Romania; adrian.teodoru@ulbsibiu.ro; 10 Department of Legal Medicine, “Iuliu Hațieganu” University of Medicine and Pharmacy, 400006 Cluj-Napoca, Romania

**Keywords:** HLA, MHC, SARS-CoV-2, COVID-19, Romania, Republic of Moldova

## Abstract

In this study, we aimed to investigate whether specific HLA alleles found in patients from Romania and the Republic of Moldova were associated with the severity of COVID-19 infection and its associated mortality. We analyzed the HLA alleles at the -A, -B, -C, -DRB1, and -DQB1 loci in a cohort of 130 individuals with severe and extremely severe forms of COVID-19, including 44 individuals who died. We compared these findings to a control group consisting of individuals who had either not been diagnosed with COVID-19 or had experienced mild forms of the disease. Using multivariate logistic regression models, we discovered that the B*27 and B*50 alleles were associated with an increased susceptibility to developing a severe form of COVID-19. The A*33 and C*15 alleles showed potential for offering protection against the disease. Furthermore, we identified two protective alleles (A*03 and DQB1*02) against the development of extremely severe forms of COVID-19. By utilizing score statistics, we established a statistically significant association between haplotypes and disease severity (*p* = 0.021). In summary, this study provides evidence that HLA genotype plays a role in influencing the clinical outcome of COVID-19 infection.

## 1. Introduction

The 2019 coronavirus disease (COVID-19) was officially declared a pandemic by the World Health Organization (WHO) in 2020. This disease, caused by the severe acute respiratory syndrome coronavirus 2 (SARS-CoV-2), manifests with a broad spectrum of symptoms that can range from mild to moderate and, in some cases, even severe, potentially resulting in death [1]. Although threats related to infectious diseases remain one of the major global challenges, the mortality associated with them has decreased in recent years. Due to the large number of deaths, SARS-CoV-2 has recently been added to the list of problematic emerging pathogens of the 21st century [2].

The impact of COVID-19 was different, both at the national level and in terms of individual patients. At the patient level, it has been shown that the risks of developing more severe forms of the disease are found in men, older patients, those with significant pre-existing medical conditions, and those with a high body mass index [3]. At the national level, analyzing the impact of COVID-19 on a population is a highly complex phenomenon that depends on various factors such as the environment, geographic region, and socioeconomic conditions. Additionally, the presence of symptoms and the severity of the disease are influenced significantly by the immunogenetic factors of the host. Throughout the course of evolution, microorganisms can impose selective pressure on humans, resulting in genetically determined variations among populations in terms of their resistance or susceptibility to infectious pathogens [4]. Due to COVID-19 being caused by a recently identified virus in humans, there is a scarcity of data regarding the genetic risk factors in the host population.

During the pandemic, variations were observed in the number of confirmed COVID-19 cases not only between countries but also within different regions of the same country [5]. Furthermore, the severity of the infection can vary widely, ranging from being asymptomatic to progressing into a severe acute respiratory syndrome, with certain areas experiencing a mortality rate of 2.6% [6].

In this regard, researchers are actively investigating the potential mechanisms involved in the immune system response against SARS-CoV-2, including the role of the major histocompatibility complex (MHC). The MHC, located on the short arm of chromosome 6, represents the most complex genetic system within the human genome. It encompasses genes that encode human leukocyte antigens (HLA), which are cell membrane proteins responsible for immune system regulation. The HLA system comprises approximately 27,000 alleles classified into three gene classes: class I, II, and III. Among these gene classes, HLA class I (A, B, C) and class II (DR, DQ, DP) are particularly vital in human immunological responses, including antigen presentation to T lymphocytes and the identification of self and non-self proteins. Polymorphisms in HLA class I and II genes are recognized as influential alleles in the susceptibility to autoimmune diseases [7] and are involved in the fine regulation of acquired immune responses [8].

Various HLA alleles exhibit distinct peptide binding repertoires, leading to associations between different infectious diseases and specific HLA antigens. Consequently, this results in diverse humoral and cellular immune responses targeting various viral epitopes. These HLA-associated immune responses have the potential to significantly impact the T-cell immune response [9]. Thus, different HLA genetic polymorphisms have been linked to the susceptibility and progression of various infectious diseases. Some examples include hepatitis B (HBV) [10], hepatitis C (HCV) [11], dengue [12], influenzas [13,14], tuberculosis [15], Human Immunodeficiency Virus (HIV) infection [16], or malaria [17]. Regarding coronaviruses, specific HLAs have been associated with coronavirus infections, including those following the initial SARS epidemic in East Asia [18] or the Middle East Respiratory Syndrome (MERS) outbreak in 2014 in Saudi Arabia [19].

Numerous studies examining the associations between HLA and COVID-19 have emerged since the start of the pandemic. These studies have focused on small cohorts from different regions of the world, particularly in heavily affected areas. As a result, research findings have been published in countries or geographical regions such as China [20,21], Egypt [22], Italy [23,24], Japan [25], Israel [26], Brazil [27], Russia [28], Greece [29], Iran [30], Spain [31], or USA [32]. Most of these studies provided weak or conflicting results and required further validation due to relatively small sample sizes. However, in several of these studies, associations were found between the infection and/or the progression of COVID-19 and HLA alleles, indicating that the genetic profile of the respective populations may influence this infection. To the best of our knowledge, no such study investigating the association between HLA and COVID-19 has been conducted in the population of Romania or the Republic of Moldova so far.

The aim of this study was to determine the frequencies of HLA class I (A, B, C) and class II (DRB1, DQB1) alleles in patients from Romania and the Republic of Moldova who had severe and extremely severe forms of COVID-19 and compare them with those found in a control group. The case group was divided into patients who had severe forms but recovered and patients who died from COVID-19. The control group comprised individuals who either had not been diagnosed with COVID-19 or had very mild forms of the disease.

## 2. Results

### 2.1. Characterization of the Study Group 

The median age for all the participants in our study was 42 (IQR 34-61), ranging from 18 to 94. The age of the severe group was statistically significantly higher than that of the control group. The distribution based on sex was similar. The general characteristics of the participants in the study are shown in Table 1.

Within the severe group, the deceased participants were significantly older; were more frequently male; had comorbidities like cardiovascular, hepatic, renal, and cancers; and were less frequently vaccinated (Table 2). Furthermore, the deceased participants were significantly more frequently present in the intensive care units and more frequently needed oxygen therapy (albeit the difference was non-significant). 

Furthermore, the severe group was divided into participants who were deceased or had an ICU stay and those who were not in these situations. The participants who deceased or had an ICU stay were significantly older, more frequently males, and had more comorbidities (pulmonary, cardiovascular, hepatic, renal, cancers), but fewer were vaccinated compared to the others (Table 3). 

From another perspective, within the severe group, we looked for the need for oxygen therapy. The participants who needed oxygen therapy were significantly older and had more comorbidities (pulmonary, cardiovascular, diabetes), but fewer were vaccinated compared to the others (Table 4). 

### 2.2. Allele Analysis 

In the univariate analysis, concerning the comparison between the severe and control groups, we found the following alleles that increased the odds of severe disease: A*02, B*27, B*50 (Table 5, Supplementary Tables S1–S5) (statistically significant). Next, we found the following alleles that decreased the odds of severe disease: A*33, B*40, B*41, B*58, C*15, DRB1*15, DQB1*06 (statistically significant). A multivariate logistic regression was then performed, where we adjusted the significant alleles for age and sex. After adjustment, the following alleles remained significant: A*33, B*27, B*50, C*15. 

Concerning the severe group, we found the following alleles that increased the odds of death: A*30, B*18, C*07, DRB1*11 (Table 6, Supplementary Tables S6–S10) (statistically significant). No alleles showed statistically significant decreases in the odds of death. Next, we adjusted these alleles for age, sex, number of comorbidities, and vaccination in a multivariate logistic regression model, and none of them remained significant, although A*30 was closer to the limit of significance (Table 6, Supplementary Tables S21–S24). 

Within the severe group, we found the following alleles that increased the odds of death or ICU: DQB1*06 (Table 7, Supplementary Tables S11–S15) (statistically significant). Moreover, the following alleles decreased the odds of death or ICU: A*03 and C*04 (statistically significant). For these alleles, we fit a multivariate logistic regression model, adjusted for age, sex, number of comorbidities, and vaccination, and only A*03 remained statistically significant (Table 7, Supplementary Tables S25–S27). 

Concerning the severe group, we found the following alleles that decreased the odds of oxygen therapy: A*03, DQB1*02 (Table 8, Supplementary Tables S16–S20) (statistically significant). No alleles increased the odds of oxygen therapy. We introduced these alleles in multivariate logistic regression models adjusted for age, sex, number of comorbidities, and vaccination, and both remained statistically significant (Table 8, Supplementary Tables S28 and S29).

### 2.3. Haplotype Analysis 

**Figure 1:**
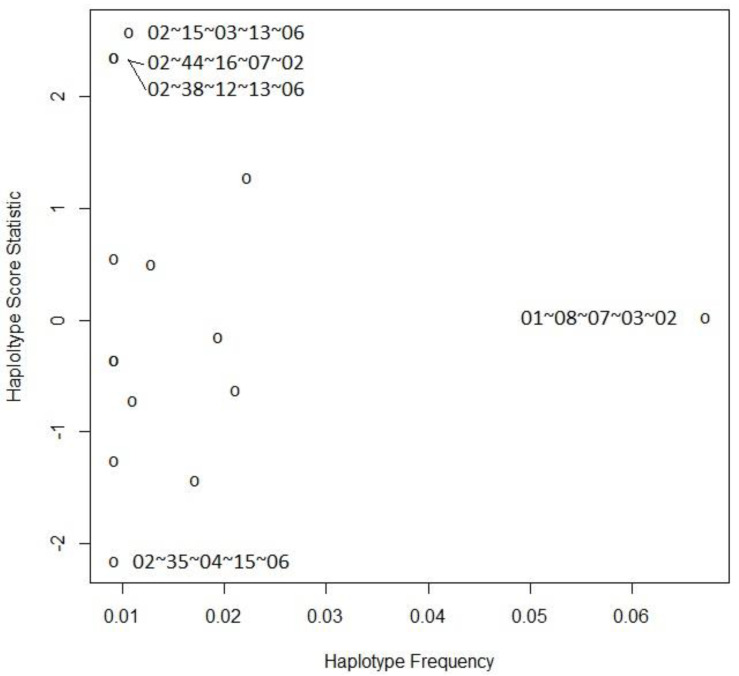
Haplotype statistics–score vs. frequency for the association between disease severity and haplotypes.

The description of the most frequently observed HLA haplotypes in the severe and control groups is presented in Table 9. We found a statistically significant association between haplotypes and severity in the dominant model using the score statistics, *p* = 0.021 (global test). The specific haplotype scores and frequencies are shown in Table 10 and Figure 1.

## 3. Discussion

It is widely recognized that HLA molecules play a crucial role in presenting viral proteins to T lymphocytes, which is essential for an effective immune response and the subsequent elimination of the pathogen. This immunogenetic response, determined by the HLA genotype, can influence the susceptibility to and/or severity of COVID-19 [33]. 

The understanding of variations in infection rates among different geographic regions remains incomplete. Therefore, it is crucial to conduct detailed analyses of allele frequencies and haplotypes within diverse population groups. This is essential because the impact of COVID-19 on a population is a complex phenomenon, and the response to the viral agent can vary based on the genetic diversity within the population. 

The current study focused on a population group consisting of 65 patients from Romania and an equal number of patients from the Republic of Moldova. This selection was made due to the ethnic composition of the population in the Republic of Moldova, which predominantly comprises “Moldovans” (75.1% according to the last census), followed in proportion by those declared “Romanians” (7%) [34]. As known from history, Moldovans originate from the Romanian population in Romania [35]. Therefore, the study was conducted on a cohort of 130 individuals of Romanian origin, the population for whom HLA association studies with COVID-19 had not been conducted before. Furthermore, there are few studies regarding the frequency of HLA alleles and haplotypes in the population of this region [36,37], and our previous studies have attempted to determine correlations between HLA alleles and certain infectious diseases or mental conditions in this population [10,38,39,40].

In this population group, the study focused exclusively on individuals who experienced severe or extremely severe forms of COVID-19. The patients were categorized into two groups: deceased and survivors. This division aimed to identify any potential associations between specific HLA alleles and death. Additionally, individuals with very severe forms of the disease (including both deceased individuals and those admitted to the ICU) were compared to others to assess the involvement of HLA alleles in the progression of COVID-19. The frequencies of alleles were also compared between individuals who required oxygen support and those who did not. 

Age has been recognized as a crucial factor in determining the severity of COVID-19 and the likelihood of adverse outcomes, including death. Once again, our study reaffirmed the significant influence of age on the severity of the disease and mortality, which has been consistently demonstrated in previous research [28,41].

When comparing the deceased individuals to the survivors in the case group, it was observed that the proportion of men among the deceased was significantly higher (*p* < 0.001). The same observation was made when comparing those with extremely severe forms (deceased and admitted to ICU) to the others in the case group. This finding is consistent with previous studies that have also reported a higher prevalence of severe COVID-19 cases among males [42,43]. 

Regarding the vaccination status, significant statistical differences (*p* < 0.001) can be observed in the severe COVID-19 group between the percentages of individuals who were vaccinated and did not die compared to those who died. The same pattern was observed when comparing individuals with extremely serious forms (deceased and admitted to the ICU) to the others from the severe group (*p* = 0.002), as well as those who required oxygen compared to those who did not (*p* = 0.006). Only one person was vaccinated in the group of individuals who died, adding further evidence to the vaccine’s effectiveness in preventing death and serious forms of COVID-19. 

In this regional study, our findings revealed that certain alleles are associated with an increased risk of infection, disease progression, and associated mortality, while others may act as protective factors against this disease. While the results presented in studies conducted on other population groups have shown significant variations, certain findings from our study align with those reported in previous research.

When comparing the case group with the control group using the multivariate logistic regression method, it was observed that two HLA-B alleles (B*27 and B*50) were associated with a risk factor for serious illness. Conversely, alleles A*33 and C*15 exhibited a protective role in the context of COVID-19 severity (Table 5). 

Thus, in our study, the B*27 allele showed a statistically significant difference in frequencies between individuals with severe forms of COVID-19 and the control group (OR 4.63 (95% CI 1.57–13.78), *p* = 0.005). This indicates that it is a risk factor for serious illness. This finding is consistent with a study conducted in Italy, where a significant association was found between the HLA-B*27:07 allele and the severity of the disease [44].

Patients who carried the B*50 allele had a 7.94-fold higher risk of developing the severe form of COVID-19 compared to those who did not possess this allele (95% CI 1.25–70.14, *p* = 0.037). A study conducted in Iran also reported a higher frequency of this allele in the case group (patients with severe symptoms) compared to the control group (20% vs. 10%) [45].

The A*30 allele was identified in our study as a risk factor for mortality, closer to the limit of significance (Table 6). Another study conducted in the United States found that the A*30:02 allele is one of the predisposing alleles associated with an elevated risk of COVID-19 in the African-American population [46]. A study conducted in Sardinia revealed that the three-locus haplotype A*30:02~B*14:02~C*08:02 was more frequently observed in a group of 182 COVID-19 patients compared to a control group of healthy individuals. Additionally, this haplotype was strongly correlated with disease severity [47].

In the current study, the A*03 allele exhibited a statistically significant difference in frequencies between individuals with very severe forms of COVID-19 (deceased/admitted to the ICU) compared to others (OR 0.14 (95%CI 0.02–0.77), *p* = 0.036) (Table 7). Additionally, a significant difference in frequencies was observed between those who required oxygen and those who did not (OR 0.26 (95%CI 0.07–0.85), *p* = 0.032) (Table 8), indicating that the A*03 allele acts as a protective factor against the development of severe symptoms. Accordingly, in a Russian study, the HLA-A*03:01 allele was found to be over-represented in a low-risk group for severe COVID-19 disease, while it was completely absent in the high-risk group [48]. Additionally, in a meta-analysis, a protective effect of the HLA-A*03 allele was indicated [49]. Contrarily, a Spanish study reported a trend toward a higher prevalence of the HLA-A*03 allele (*p* = 0.047) in COVID-19 patients compared to healthy controls. However, these *p*-values did not reach statistical significance after correction for multiple comparisons [50].

The DQB1*02 allele has been identified as a protective factor, significantly reducing the odds of requiring oxygen therapy (OR 0.31 (95%CI 0.13–0.70), *p* = 0.006). A pilot study conducted in Mexico revealed that the frequency of HLA-DQB1*02 is lower in severe COVID-19 patients [51].

In our study, we observed a statistically significant association between haplotypes and the severity of the disease (*p* = 0.021, global test). The most prevalent haplotype identified in both the severe and control groups was A*01~B*08~C*07~DRB1*03~DQB1*02 (Table 9). This haplotype aligns with the most frequently found haplotype in our previous study conducted on the population of Romanian origin in Transylvania [37]. To differentiate between the two groups more accurately, further studies employing high-resolution tests would be necessary.

The HLA-A*01:01~B*08:01~C*07:01~DRB1*03:01~DQA1*05:01~DQB1*02:01 haplotype is known as the 8.1 ancestral haplotype and is commonly found in Caucasoid populations. This haplotype has been extensively studied and has been associated with various autoimmune disorders and immune-related conditions in different populations. The literature reports that individuals carrying the 8.1 ancestral haplotypes have an increased risk of developing specific autoimmune disorders compared to those without these alleles [52]. Since this haplotype is associated with susceptibility to numerous autoimmune diseases, it can be inferred that carriers of this haplotype may exhibit altered regulation of the cell-mediated immune response, potentially leading to more severe clinical manifestations.

A study conducted in Italy compared the regional frequencies of the most common Italian haplotypes from the Italian Bone Marrow Donor Registry. The study found that the haplotype A*01:01~B*08:01~C*07:01~DRB1*03:01, which is one of the most prevalent in the Italian population, exhibited a significant positive correlation (suggestive of susceptibility) with both COVID-19 incidence and mortality [53]. Additionally, the Italian study revealed that the haplotype A*02:01~B*18:01~C*07:01~DRB1*11:04, also among the most common in the population, displayed a significant negative correlation (suggestive of protection) with both COVID-19 incidence and mortality.

In our study, the haplotypes in positions 3, 4, 5, 6, 7, and 10 within the group with severe forms are not present among the top 10 haplotypes in terms of frequency in the control group. This indicates that individuals carrying these haplotypes may be predisposed to severe forms of COVID-19. A study conducted in Sardinia found that the haplotype A*02:05~B*58:01~C*07:01~DRB1*03:01 has a protective effect against SARS-CoV-2 infection within the population of this region [47]. In the present study, we identified a similar haplotype, A*02~B*41~C*07~DRB1*03~DQB1*02, ranking eighth in the control group and absent among the top ten in the severe forms group. This finding suggests its potential protective effect.

Despite the promising results, this study has several limitations. Firstly, the cohort consisted of a relatively small number of patients, which may limit the power and generalizability of the findings. Secondly, HLA typing was performed at a low resolution, potentially missing more specific associations. Since it is an observational study, no strong statement concerning the cause-effect relation could be sustained. Moreover, confounding is a possibility with this study design. Nevertheless, we adjusted for known confounders in multiple regressions. Of course, residual confounding can still be an issue. Additionally, the specific variant of SARS-CoV-2 was not determined in this study. However, given that the patients were recruited between April 2020 and March 2022, it is likely that most of them were infected with prevalent variants during that period in the specific region, such as the Alpha variant (B.1.1.7) and Delta variant (B.1.617.2) [54]. These variants are known to be associated with increased disease severity and have had a significant impact on infection and mortality rates in some countries [55]. The Omicron variant generally causes mild clinical symptoms and has resulted in fewer severe cases and deaths compared to earlier variants [56]. Another limitation of the study can be considered the inclusion in the control group of both healthy volunteers and patients with mild COVID-19, but it was considered that all these persons were protected from developing a serious form of the disease.

Due to the high level of polymorphism in HLA genes, it is important to interpret the results of this study with caution. The investigation of multiple alleles increases the risk of identifying a seemingly “significant” allele by chance alone. Therefore, it is crucial to conduct future studies with larger sample sizes to validate and confirm these findings. Additionally, further research is needed to explore whether other HLA loci play a role in influencing SARS-CoV-2 infection and the progression of COVID-19 in this specific population. Furthermore, it is advisable to investigate these initial findings using high-resolution assays.

Considering the continuous evolution of SARS-CoV-2 variants, it is crucial to evaluate the importance of background immunogenetic characteristics in diverse populations. Such an assessment is vital to facilitate the development of targeted and more effective early intervention strategies for future outbreaks. Understanding the impact of HLA allele variability on T cell responses is crucial for comprehending immunogenic mechanisms in both natural immunity and vaccine development. In silico analyses have consistently identified HLA molecules as relevant factors in SARS-CoV-2 susceptibility, the heterogeneity of clinical outcomes, and important targets for vaccine development [57,58,59,60]. It has also been shown that patients with COVID-19 exhibited significant differences in blood-lymphocyte kinetics, and these changes were linked to the disease severity and final outcomes [61]. It has been suggested that patients with the most severe forms of the disease have an increased functionality of T cells recognizing specific antigens presented by one of their HLAs, which could be specifically studied using the predicted epitopes [59].

The present study offers partial insights, which can guide future investigations and contribute to the ongoing vaccination efforts. By generating conclusive answers, this analysis has the potential to significantly aid the development of personalized treatments, diagnostics, and vaccines. An enhanced understanding of these mechanisms could prove to be the greatest advantage in the race to develop more effective drugs and vaccines against COVID-19.

## 4. Materials and Methods

### 4.1. Study Design, Setting, and Participants

A cross-sectional design study was undertaken. In the case group, a total of 130 patients with severe forms of COVID-19 were included in the study between April 2020 and March 2022. Out of the total number of patients, 65 (50%) were from Romania, while the remaining patients were from the Republic of Moldova, residents of each country.

Among the patients from Romania, 28 consecutive individuals who died with a COVID-19 diagnosis, confirmed post-mortem at the Legal Medicine Institute (IML) Cluj-Napoca, were included in the case group. The diagnosis was made using the real-time polymerase chain reaction (RT-PCR) technique on a necroptic lung fragment, following the observation of specific signs of COVID-19 during the autopsy. The other participants from Romania were consecutive patients admitted to the intensive care unit (ICU) at the Emergency Clinical County Hospital in Târgu Mureș and confirmed to have COVID-19 through RT-PCR. Out of these patients, 16 died in the subsequent days. Therefore, the group from Romania consisted of 44 deceased individuals, accounting for 33.84% of the total.

From the Republic of Moldova, a total of 65 patients were included in the study. None of these patients died during the study period. Some had severe forms of COVID-19 requiring long hospitalization, while others had respiratory insufficiency, which was defined as the need for supplemental oxygen and/or mechanical ventilation without being admitted to the ICU, and the other very serious forms of COVID-19 (admitted to the ICU).

The criteria respected by clinicians for the management of COVID-19 patients (respectively, for ICU admission and intubation) were based on the requirements established at the national level in the two countries (Romania and the Republic of Moldova), based on the criteria and recommendations made at the European level by the CDC, respecting the data provided by the WHO [62,63].

Additionally, HLA profile data from a control group of 172 consecutive unrelated subjects who were not diagnosed with COVID-19 or had very mild forms of the disease were obtained. These individuals were participants in paternity tests at IML Cluj-Napoca, and their data were collected retrospectively.

This study was approved by the Ethics Committee of the “Iuliu Hațieganu” University of Medicine and Pharmacy, Cluj-Napoca, Romania (AVZ 67/11 March 2022), the Ethics Committee of the Emergency Clinical County Hospital, Târgu Mureș, Romania (Ad 3735/4 February 2022) and the Ethics Committee of the State University of Medicine and Pharmacy, Chișinău, Republic of Moldova (No. 1/14 June 2022). All participants enrolled signed the informed consent, and all results obtained were kept anonymous. For deceased persons, informed consent for genetic testing was granted by their relatives.

### 4.2. Variables

The following data were collected from all individuals included in the case group: age, sex, place of origin (urban/rural), socioeconomic status (good/medium/low), presence of chronic diseases, how many confirmed infections with the SARS-CoV-2 virus they have, vaccination status against COVID-19, and symptoms experienced. For deceased individuals, the data were provided by their relatives. From the control group, we had access to the age and gender of the participants.

### 4.3. DNA Extraction

Post-mortem, 2 mL of blood was collected from the right ventricle of each deceased patient using a vial containing ethylenediaminetetraacetic acid (EDTA). The DNA extraction process was carried out using the EPICENTRE MasterPureTM Complete DNA and RNA Purification Kit (Illumina Company in Madison, WI, USA). The extraction was performed following the manufacturer’s instructions, specifically the blood protocol.

From the patients who were still alive at the time of sample collection, 2 mL of peripheral venous blood were collected using vials containing EDTA. DNA extraction from these samples was performed using the Maxwell RSC Whole Blood DNA Kit (Promega Corporation, Madison, WI, USA). The extraction process followed the manufacturer’s instructions.

DNA concentration and purity were determined using a Pearl nanophotometer (Implen GmbH, Munich, Germany). For HLA typing, DNA samples with an A280/A260 ratio within the range of 1.8 ± 10% were considered suitable. Any samples that did not meet this criterion were subjected to purification using the EPICENTRE MasterPureTM Complete DNA and RNA Purification Kit.

### 4.4. HLA Typing

For each individual included in the study, five genes were analyzed: HLA-A, HLA-B, and HLA-C from HLA class I, and HLA-DRB1 and HLA-DQB1 from HLA class II. Diluted DNA with a concentration of 1 ng/µL was used for HLA low-resolution molecular typing. This typing was based on the sequence-specific priming polymerase chain reaction (SSP-PCR) molecular technique, using two commercial kits: HLA-FluoGene ABC (Innotrain Diagnostik GmbH, Kronberg, Germania) and HLA-FluoGene DRDQ (Inno-train Diagnostik GmbH, Kronberg, Germania). The DNA amplification process was conducted on a G-Storm thermal cycler (Gene Technologies Ltd., Essex, UK). The FluoVista Analyzer (Inno-train Diagnostik GmbH, Kronberg, Germany) was used to detect the fluorescence signals of the PCR products. The FluoGene analysis software (Innotrain Diagnostik GmbH, Kronberg, Germania) automatically calculated the endpoint fluorescence of the various fluorochromes before and after PCR [64].

### 4.5. Statistical Analysis

We described categorical data using counts and percentages and quantitative data that did not follow the normal distribution using medians and the 25 and 75 percentiles (IQR). A comparison of the two independent groups concerning categorical data was performed using the chi-squared or Fisher exact tests. A comparison of the two independent groups concerning continuous data that did not follow the normal distribution was performed using the Wilcoxon rank-sum test. To assess the strength of the association between specific HLA alleles and the outcomes of interest, we used univariate logistic regressions predicting the binary grouping variable (e.g., severity, death, death or ICU therapy, oxygen therapy), followed by multivariate logistic regressions. Within the multivariate logistic regression models, along with the HLA allele variable, we adjusted for several variables known to be associated with the dependent variable. The number of variables in the models was chosen so that it did not surpass the rule of thumb of 10 cases per degree of freedom within the smallest category of the dependent variable to prevent overfitting. The Hosmer and Lemeshow test was used to assess the goodness-of-fit of the model. For multivariate models, the multicollinearity assumption was assessed with variance inflation factors. All regression models were presented by odds ratios, confidence intervals, and *p*-values. The haplotype frequencies were computed for severe and control groups. To assess the association between HLA haplotypes and the severity of disease, we used the score tests [65]. All haplotype analyses were computed in the haplo.stats R package version 1.9.3 [66].

## 5. Conclusions

In conclusion, this study has provided the first evidence that HLA polymorphisms can impact the rate of SARS-CoV-2 infection and disease progression within the population of this region.

In our study, we identified two HLA-B alleles (B*27 and B*50) that were associated with an increased susceptibility to developing a severe form of the disease. Additionally, we found two HLA alleles (A*33 and C*15) that showed potential for providing protection against the disease. Furthermore, we identified two protective alleles (A*03 and DQB1*02) against the development of extremely severe forms of COVID-19, including death or the need for ICU care or oxygen support. We discovered a statistically significant association between haplotypes and the severity of the infection using score statistics, with a *p*-value of 0.021 (global test).

These findings, combined with studies conducted in other parts of the world, may partially elucidate the varying global spread of the virus and the observed discrepancies in disease severity and mortality rates. Moreover, the identification of these alleles enables the identification of individuals at higher risk of infection or developing severe forms of the disease. The identification of HLA antigens with a strong affinity for binding viral peptides holds promise in formulating targeted vaccination strategies for specific populations.

## Figures and Tables

**Table 1:** Participants’ characteristics.

Group	Control (n = 169)	Severe (n = 130)	*p*-Value
Age (years), median (IQR ^1^)	35 (29–40)	63 (53–70.75)	**<0.001**
Sex (F), no (%)	82 (48.52)	67 (51.54)	0.605

^1^ IQR, interquartile range; bold text indicates a statistically significant difference between the two groups.

**Table 2:** Patients’ characteristics by deceased status in the severe COVID-19 group.

Characteristics	Not Deceased (n = 86)	Deceased (n = 44)	*p*-Value
Age (years), median (IQR ^1^)	60.5 (51–67)	67 (59.5–77)	**<0.001**
Sex			
Female, n (%)	57 (66.28)	10 (22.73)	**<0.001**
Male, n (%)	29 (33.72)	34 (77.27)	
Comorbidities			
Pulmonary, n (%)	8 (9.3)	6 (13.64)	0.552
Cardiovascular, n (%)	9 (10.47)	32 (72.73)	**<0.001**
Obesity, n (%)	6 (4.11)	10 (11.63)	**0.029**
Diabetes, n (%)	38 (26.03)	10 (11.63)	**0.009**
Hepatic, n (%)	6 (6.98)	13 (29.55)	**<0.001**
Renal, n (%)	1 (1.16)	14 (31.82)	**<0.001**
Cancers, n (%)	1 (1.16)	2 (4.55)	0.264
Vaccinated, n (%)	28 (40.58)	1 (4)	**<0.001**
Oxygen therapy, n (%)	49 (56.98)	23 (71.88)	0.14
ICU ^2^, n (%)	26 (31.33)	18 (56.25)	**0.014**

^1^ IQR, interquartile range; ^2^ ICU, intensive care unit; bold text indicates a statistically significant difference between the two groups.

**Table 3:** Patients’ characteristics by deceased or intensive care unit stay status in the severe COVID-19 group.

Characteristics	Not Deceased/ICU ^1^ (n = 57)	Deceased/ICU ^1^ (n = 73)	*p*-Value
Age (years), median (IQR ^2^)	57 (46–63)	67 (59–77)	**<0.001**
Sex			**<0.001**
Female, n (%)	42 (73.68)	25 (34.25)	
Male, n (%)	15 (26.32)	48 (65.75)	
Comorbidities			
Pulmonary, n (%)	2 (3.51)	12 (16.44)	**0.018**
Cancers, n (%)	1 (1.75)	40 (54.79)	**<0.001**
Cardiovascular, n (%)	1 (1.75)	40 (54.79)	**<0.001**
Obesity, n (%)	0 (0)	16 (11.27)	**<0.001**
Diabetes, n (%)	24 (26.67)	24 (16.9)	0.074
Hepatic, n (%)	2 (3.51)	17 (23.29)	**0.002**
Renal, n (%)	0 (0)	15 (20.55)	**<0.001**
Vaccinated, n (%)	23 (44.23)	6 (14.29)	**0.002**

^1^ ICU, intensive care unit; ^2^ IQR, interquartile range; bold text indicates a statistically significant difference between the two groups.

**Table 4:** Patients’ characteristics by oxygen therapy in the severe COVID-19 group.

Characteristics	Without Oxygen (n = 46)	With Oxygen (n = 72)	*p*-Value
Age (years), median (IQR ^1^)	53 (42.5–62.75)	64 (59.75–73.5)	**<0.001**
Sex			0.077
Female, n (%)	30 (65.22)	35 (48.61)	
Male, n (%)	16 (34.78)	37 (51.39)	
Comorbidities			
Pulmonary, n (%)	1 (2.17)	12 (16.67)	**0.014**
Cancers, n (%)	0 (0)	2 (2.78)	0.52
Cardiovascular, n (%)	8 (17.39)	25 (34.72)	**0.041**
Obesity, n (%)	2 (2.86)	12 (8.7)	0.147
Diabetes, n (%)	10 (14.29)	38 (27.54)	**0.032**
Hepatic, n (%)	6 (13.04)	9 (12.5)	0.931
Renal, n (%)	6 (13.04)	5 (6.94)	0.335
Vaccinated, n (%)	20 (46.51)	9 (19.15)	**0.006**
Deceased, n (%)	9 (19.57)	23 (31.94)	0.14

^1^ IQR, interquartile range; bold text indicates a statistically significant difference between the two groups.

**Table 5:** Simple and multiple logistic regressions predicting severe group compared to control, based on HLA alleles, adjusted for age and sex.

HLA ^1^ Allele n ^2^ (%)	Severe (n = 260)	Control (n = 338)	OR ^3^ Unadjusted (95% CI ^4^)	*p*-Value	OR Adjusted (95% CI)	*p*-Value
**A**	**n = 260**	**n = 318**				
A*02	77 (29.62)	70 (22.01)	1.49 (1.02–2.17)	**0.037**	1.6 (0.88–2.9)	0.121
A*33	1 (0.38)	11 (3.46)	0.11 (0.01–0.84)	**0.010**	0.03 (0–0.3)	**0.006**
**B**	**n = 260**	**n = 318**				
B*27	22 (8.46)	14 (4.4)	2.01 (1.01–4.01)	**0.045**	4.63 (1.57–13.78)	**0.005**
B*40	10 (3.85)	26 (8.18)	0.45 (0.21–0.95)	**0.032**	0.48 (0.12–1.71)	0.278
B*41	1 (0.38)	9 (2.83)	0.13 (0.02–1.05)	**0.027**	0.33 (0.01–5.07)	0.526
B*50	10 (3.85)	2 (0.63)	6.32 (1.37–29.11)	**0.007**	7.94 (1.25–70.14)	**0.037**
B*58	0 (0)	6 (1.89)	0 (0–NC ^5^)	**0.035**	0 (NA–3.8 × 10^30^)	0.982
**C**	**n = 260**	**n = 318**				
C*15	8 (3.08)	25 (7.86)	0.37 (0.16–0.84)	**0.014**	0.13 (0.03–0.53)	**0.004**
**DRB1**	**n = 260**	**n = 304**				
DRB1*15	17 (6.54)	37 (12.17)	0.5 (0.28–0.92)	**0.023**	0.88 (0.33–2.17)	0.793
**DQB1**	**n = 260**	**n = 304**				
DQB1*06	33 (12.69)	63 (20.72)	0.56 (0.35–0.88)	**0.011**	0.69 (0.32–1.41)	0.314

^1^ HLA, human leukocyte antigen; ^2^ n, allele number; ^3^ OR, odds ratio; ^4^ CI, confidence interval; ^5^ NC, cannot be computed, bold text indicates a statistically significant difference between the two groups.

**Table 6:** Simple and multiple logistic regressions predicting deceased within the severe group, based on HLA alleles, adjusted for age, sex, number of comorbidities, and vaccination.

HLA ^1^ Allele	Deceased (n ^2^ = 88)	Not Deceased (n = 172)	OR ^3^ Unadjusted (95% CI ^4^)	*p*-Value	OR Adjusted (95% CI)	*p*-Value	Hosmer Lemeshow Test	AUROC ^5^ (95% CI)
A*30	7 (7.95)	3 (1.74)	4.87 (1.23–19.32)	**0.034**	14.05 (0.59–238.42)	0.073	0.430	0.94 (0.90–0.97)
B*18	16 (18.18)	12 (6.98)	2.96 (1.33–6.58)	**0.006**	1.46 (0.36–5.98)	0.597	0.032	0.94 (0.90–0.97)
C*07	33 (37.5)	39 (22.67)	2.05 (1.17–3.58)	**0.011**	1.54 (0.49–4.85)	0.456	0.230	0.94 (0.90–0.97)
DRB1*11	25 (28.41)	30 (17.44)	1.88 (1.02–3.45)	**0.040**	0.89 (0.21–3.56)	0.874	0.188	0.94 (0.90–0.97)

^1^ HLA, human leukocyte antigen; ^2^ n, allele number; ^3^ OR, odds ratio; ^4^ CI, confidence interval; ^5^ AUROC, area under the receiver operating characteristic; bold text indicates a statistically significant difference between the two groups.

**Table 7:** Multiple logistic regressions predicting deceased or ICU stay within severe group, based on HLA alleles, adjusted for age, sex, number of comorbidities, and vaccination.

HLA ^1^ Allele	Deceased/ICU ^2^ (n ^3^ = 146)	Not Deceased/ICU ^2^ (n = 114)	OR ^4^ Unadjusted (95% CI ^5^)	*p*-Value	OR Adjusted (95% CI)	*p*-Value	Hosmer Lemeshow Test	AUROC ^6^ (95% CI)
A*03	10 (6.85)	17 (14.91)	0.42 (0.18–0.96)	**0.034**	0.14 (0.02–0.77)	**0.036**	0.123	0.92 (0.88–0.95)
C*04	16 (10.96)	26 (22.81)	0.42 (0.21–0.82)	**0.010**	0.48 (0.15–1.5)	0.218	0.116	0.91 (0.87–0.95)
DQB1*06	24 (16.44)	9 (7.89)	2.3 (1.02–5.16)	**0.040**	3.2 (0.95–11.46)	0.065	0.519	0.92 (0.88, 0.95)

^1^ HLA, human leukocyte antigen; ^2^ ICU, intensive care unit; ^3^ n, allele number; ^4^ OR, odds ratio; ^5^ CI, confidence interval; ^6^ AUROC, area under the receiver operating characteristic; bold text indicates a statistically significant difference between the two groups.

**Table 8:** Multiple logistic regressions predicting oxygen therapy within the severe group, based on HLA alleles, adjusted for age, sex, number of comorbidities, and vaccination.

HLA ^1^ Allele	With Oxygen (n ^2^ = 144)	Without Oxygen (n = 92)	OR ^3^ Unadjusted (95% CI ^4^)	*p*-Value	OR Adjusted (95% CI)	*p*-Value	Hosmer Lemeshow Test	AUROC ^5^ (95% CI)
A*03	11 (7.64)	15 (16.3)	0.42 (0.19–0.97)	**0.038**	0.26 (0.07–0.85)	**0.032**	0.933	0.82 (0.76–0.88)
DQB1*02	28 (19.44)	31 (33.7)	0.47 (0.26–0.86)	**0.014**	0.31 (0.13–0.7)	**0.006**	0.89	0.83 (0.77–0.89)

^1^ HLA, human leukocyte antigen; ^2^ n, allele number; ^3^ OR, odds ratio; ^4^ CI, confidence interval; ^5^ AUROC, area under the receiver operating characteristic; bold text indicates a statistically significant difference between the two groups.

**Table 9:** The most frequent HLA haplotypes in the severe group and control group.

Severe						Control					
A	B	C	DRB1	DQB1	Probability	A	B	C	DRB1	DQB1	Probability
01	08	07	03	02	0.065	01	08	07	03	02	0.070
02	18	07	11	03	0.025	01	40	15	14	05	0.025
02	15	03	13	06	0.023	03	35	04	01	05	0.025
02	38	12	13	06	0.019	03	35	04	11	03	0.018
02	44	16	07	02	0.019	02	35	04	15	06	0.018
24	35	04	11	03	0.019	02	18	07	11	03	0.018
02	18	07	16	05	0.019	02	52	12	15	05	0.014
03	35	04	01	05	0.017	02	41	07	03	02	0.014
03	07	07	11	03	0.015	03	07	07	11	03	0.011
26	38	12	14	05	0.015	02	51	14	11	03	0.011

**Table 10:** Haplotype statistics; score vs. frequency for the association between disease severity and haplotypes.

A	B	C	DRB1	DQB1	Hap-Freq	Hap-Score	*p*-Value
02	35	04	15	06	0.00919	−2.15944	**0.03082**
01	40	15	14	05	0.01717	−1.43421	0.15151
02	41	07	03	02	0.00919	−1.25580	0.20919
11	52	12	15	06	0.01103	−0.71707	0.47333
03	35	04	01	05	0.02108	−0.62201	0.53393
02	52	12	15	05	0.00919	−0.35216	0.72472
26	27	01	01	05	0.00919	−0.35215	0.72472
24	35	04	11	03	0.01946	−0.15135	0.87970
01	08	07	03	02	0.06722	0.02675	0.97866
03	07	07	11	03	0.01287	0.50167	0.61590
30	13	06	07	02	0.00919	0.55149	0.58130
02	18	07	11	03	0.02224	1.27490	0.20234
02	38	12	13	06	0.00919	2.35877	**0.01834**
02	44	16	07	02	0.00919	2.35877	**0.01834**
02	15	03	13	06	0.01070	2.58579	**0.00972**

## Data Availability

The data that support the findings of this study are available from the corresponding author upon reasonable request.

## Supplementary Materials

The following supporting information can be downloaded at: https://www.mdpi.com/article/10.3390/ijms25021326/s1.
