# Supplementary material for: The Influence of HLA Polymorphisms on the Severity of COVID-19 in the Romanian Population
Source: Int J Mol Sci. 2024 Jan 22;25(2):1326. doi: 10.3390/ijms25021326 (PMC10816224; doi:10.3390/ijms25021326)
Supplement: Supplementary file 1 [file ijms-25-01326-s001.zip › ijms-2795712-supplementary.pdf]

Contents

Comparison between severe and control groups 1
Within severe group 4
Comparison concerning deceased status 4
Comparison concerning diseased or ICU status 6
Comparisons concerning oxygen therapy 9
Multiple logistic regressions 12

Comparison between severe and control groups

Supplementary Table S1. Univariate logistic regression analysis predicting the odds of severe disease based on HLA-A alleles

Group:	Severe (n=260)	Control (n=318)	OR (95% CI), P-value
A*01 n (%)	41 (15.77)	54 (16.98)	0.92 (95% CI 0.59 - 1.43), 0.696
A*02 n (%)	77 (29.62)	70 (22.01)	1.49 (95% CI 1.02 - 2.17), 0.037
A*03 n (%)	27 (10.38)	43 (13.52)	0.74 (95% CI 0.44 - 1.24), 0.25
A*11 n (%)	17 (6.54)	30 (9.43)	0.67 (95% CI 0.36 - 1.25), 0.205
A*23 n (%)	3 (1.15)	3 (0.94)	1.23 (95% CI 0.25 - 6.12), 1
A*24 n (%)	35 (13.46)	36 (11.32)	1.22 (95% CI 0.74 - 2), 0.435
A*25 n (%)	4 (1.54)	8 (2.52)	0.61 (95% CI 0.18 - 2.03), 0.412
A*26 n (%)	14 (5.38)	14 (4.4)	1.24 (95% CI 0.58 - 2.64), 0.584
A*29 n (%)	1 (0.38)	6 (1.89)	0.2 (95% CI 0.02 - 1.68), 0.136
A*30 n (%)	10 (3.85)	13 (4.09)	0.94 (95% CI 0.4 - 2.18), 0.882
A*31 n (%)	7 (2.69)	8 (2.52)	1.07 (95% CI 0.38 - 3), 0.894
A*32 n (%)	13 (5)	12 (3.77)	1.34 (95% CI 0.6 - 2.99), 0.471
A*33 n (%)	1 (0.38)	11 (3.46)	0.11 (95% CI 0.01 - 0.84), 0.01
A*66 n (%)	1 (0.38)	2 (0.63)	0.61 (95% CI 0.06 - 6.77), 1
A*68 n (%)	5 (1.92)	7 (2.2)	0.87 (95% CI 0.27 - 2.78), 0.815
A*69 n (%)	3 (1.15)	1 (0.31)	3.7 (95% CI 0.38 - 35.79), 0.331
A*74 n (%)	1 (0.38)	0 (0)	Inf (95% CI NC - Inf), 0.45

n for groups, number of alleles; HLA, human leukocyte antigen; OR, odds ratio; CI, confidence interval; NC, cannot be computed.

Supplementary Table S2. Univariate logistic regression analysis predicting the odds of severe disease based on HLA-B alleles

Group:	Severe (n=260)	Control (n=318)	OR (95% CI), P-value
B*07 n (%)	18 (6.92)	11 (3.46)	2.08 (95% CI 0.96 - 4.48), 0.058

B*08 n (%)	26 (10)	31 (9.75)	1.03 (95% CI 0.59 - 1.78), 0.92
B*13 n (%)	8 (3.08)	8 (2.52)	1.23 (95% CI 0.46 - 3.32), 0.682
B*14 n (%)	4 (1.54)	9 (2.83)	0.54 (95% CI 0.16 - 1.76), 0.297
B*15 n (%)	13 (5)	10 (3.14)	1.62 (95% CI 0.7 - 3.76), 0.256
B*18 n (%)	28 (10.77)	29 (9.12)	1.2 (95% CI 0.7 - 2.08), 0.508
B*27 n (%)	22 (8.46)	14 (4.4)	2.01 (95% CI 1.01 - 4.01), 0.045
B*35 n (%)	30 (11.54)	54 (16.98)	0.64 (95% CI 0.39 - 1.03), 0.065
B*37 n (%)	1 (0.38)	2 (0.63)	0.61 (95% CI 0.06 - 6.77), 1
B*38 n (%)	12 (4.62)	9 (2.83)	1.66 (95% CI 0.69 - 4.01), 0.254
B*39 n (%)	6 (2.31)	5 (1.57)	1.48 (95% CI 0.45 - 4.9), 0.554
B*40 n (%)	10 (3.85)	26 (8.18)	0.45 (95% CI 0.21 - 0.95), 0.032
B*41 n (%)	1 (0.38)	9 (2.83)	0.13 (95% CI 0.02 - 1.05), 0.027
B*44 n (%)	22 (8.46)	24 (7.55)	1.13 (95% CI 0.62 - 2.07), 0.686
B*47 n (%)	1 (0.38)	2 (0.63)	0.61 (95% CI 0.06 - 6.77), 1
B*49 n (%)	4 (1.54)	3 (0.94)	1.64 (95% CI 0.36 - 7.4), 0.706
B*50 n (%)	10 (3.85)	2 (0.63)	6.32 (95% CI 1.37 - 29.11), 0.007
B*51 n (%)	17 (6.54)	33 (10.38)	0.6 (95% CI 0.33 - 1.11), 0.102
B*52 n (%)	12 (4.62)	16 (5.03)	0.91 (95% CI 0.42 - 1.97), 0.817
B*53 n (%)	1 (0.38)	0 (0)	Inf (95% CI NC - Inf), 0.45
B*55 n (%)	5 (1.92)	6 (1.89)	1.02 (95% CI 0.31 - 3.38), 1
B*56 n (%)	2 (0.77)	4 (1.26)	0.61 (95% CI 0.11 - 3.35), 0.695
B*57 n (%)	7 (2.69)	5 (1.57)	1.73 (95% CI 0.54 - 5.52), 0.347
B*58 n (%)	0 (0)	6 (1.89)	0 (95% CI 0 - NC), 0.035

n for groups, number of alleles; HLA, human leukocyte antigen; OR, odds ratio; CI, confidence interval; NC, cannot be computed.

Supplementary Table S3. Univariate logistic regression analysis predicting the odds of severe disease based on HLA-C alleles

Group:	Severe (n=260)	Control (n=318)	OR (95% CI), P-value
C*01 n (%)	12 (4.62)	22 (6.92)	0.65 (95% CI 0.32 - 1.34), 0.242
C*02 n (%)	21 (8.08)	19 (5.97)	1.38 (95% CI 0.73 - 2.63), 0.322
C*03 n (%)	20 (7.69)	22 (6.92)	1.12 (95% CI 0.6 - 2.1), 0.721
C*04 n (%)	42 (16.15)	52 (16.35)	0.99 (95% CI 0.63 - 1.54), 0.949
C*05 n (%)	6 (2.31)	7 (2.2)	1.05 (95% CI 0.35 - 3.16), 0.932
C*06 n (%)	24 (9.23)	20 (6.29)	1.52 (95% CI 0.82 - 2.81), 0.185
C*07 n (%)	72 (27.69)	69 (21.7)	1.38 (95% CI 0.94 - 2.02), 0.095
C*08 n (%)	4 (1.54)	11 (3.46)	0.44 (95% CI 0.14 - 1.39), 0.149
C*12 n (%)	39 (15)	50 (15.72)	0.95 (95% CI 0.6 - 1.49), 0.811
C*14 n (%)	3 (1.15)	6 (1.89)	0.61 (95% CI 0.15 - 2.45), 0.524

C*15 n (%)	8 (3.08)	25 (7.86)	0.37 (95% CI 0.16 - 0.84), 0.014
C*16 n (%)	9 (3.46)	11 (3.46)	1 (95% CI 0.41 - 2.45), 0.999
C*17 n (%)	0 (0)	4 (1.26)	0 (95% CI 0 - NC), 0.131

n for groups, number of alleles; HLA, human leukocyte antigen; OR, odds ratio; CI, confidence interval; NC, cannot be computed.

Supplementary Table S4. Univariate logistic regression analysis predicting the odds of severe disease based on HLA-DRB1 alleles

Group:	Severe (n=260)	Control (n=304)	OR (95% CI), P-value
DRB1*01 n (%)	28 (10.77)	26 (8.55)	1.29 (95% CI 0.74 - 2.26), 0.372
DRB1*03 n (%)	40 (15.38)	45 (14.8)	1.05 (95% CI 0.66 - 1.66), 0.847
DRB1*04 n (%)	19 (7.31)	28 (9.21)	0.78 (95% CI 0.42 - 1.43), 0.415
DRB1*07 n (%)	33 (12.69)	31 (10.2)	1.28 (95% CI 0.76 - 2.16), 0.352
DRB1*08 n (%)	6 (2.31)	1 (0.33)	7.16 (95% CI 0.86 - 59.84), 0.053
DRB1*09 n (%)	1 (0.38)	1 (0.33)	1.17 (95% CI 0.07 - 18.8), 1
DRB1*10 n (%)	3 (1.15)	4 (1.32)	0.88 (95% CI 0.19 - 3.95), 1
DRB1*11 n (%)	55 (21.15)	54 (17.76)	1.24 (95% CI 0.82 - 1.89), 0.309
DRB1*12 n (%)	1 (0.38)	5 (1.64)	0.23 (95% CI 0.03 - 1.99), 0.225
DRB1*13 n (%)	23 (8.85)	33 (10.86)	0.8 (95% CI 0.46 - 1.4), 0.426
DRB1*14 n (%)	12 (4.62)	16 (5.26)	0.87 (95% CI 0.4 - 1.88), 0.724
DRB1*15 n (%)	17 (6.54)	37 (12.17)	0.5 (95% CI 0.28 - 0.92), 0.023
DRB1*16 n (%)	22 (8.46)	23 (7.57)	1.13 (95% CI 0.61 - 2.08), 0.696

n for groups, number of alleles; HLA, human leukocyte antigen; OR, odds ratio; CI, confidence interval; NC, cannot be computed.

Supplementary Table S5. Univariate logistic regression analysis predicting the odds of severe disease based on HLA-DQB1 alleles

Group:	Severe (n=260)	Control (n=304)	OR (95% CI), P-value
DQB1*02 n (%)	68 (26.15)	71 (23.36)	1.16 (95% CI 0.79 - 1.71), 0.442
DQB1*03 n (%)	84 (32.31)	96 (31.58)	1.03 (95% CI 0.73 - 1.47), 0.853
DQB1*04 n (%)	5 (1.92)	2 (0.66)	2.96 (95% CI 0.57 - 15.39), 0.257
DQB1*05 n (%)	70 (26.92)	72 (23.68)	1.19 (95% CI 0.81 - 1.74), 0.377
DQB1*06 n (%)	33 (12.69)	63 (20.72)	0.56 (95% CI 0.35 - 0.88), 0.011

n for groups, number of alleles; HLA, human leukocyte antigen; OR, odds ratio; CI, confidence interval; NC, cannot be computed.

Within severe group

Comparison concerning deceased status

Supplementary Table S6. Univariate logistic regression analysis predicting the odds of death based on HLA-A alleles

Deceased:	Yes (n=88)	No (n=172)	OR (95% CI), P-value
A*01 n (%)	14 (15.91)	27 (15.7)	1.02 (95% CI 0.5 - 2.05), 0.965
A*02 n (%)	26 (29.55)	51 (29.65)	0.99 (95% CI 0.57 - 1.75), 0.986
A*03 n (%)	8 (9.09)	19 (11.05)	0.81 (95% CI 0.34 - 1.92), 0.625
A*11 n (%)	5 (5.68)	12 (6.98)	0.8 (95% CI 0.27 - 2.36), 0.689
A*23 n (%)	0 (0)	3 (1.74)	0 (95% CI 0 - NC), 0.553
A*24 n (%)	16 (18.18)	19 (11.05)	1.79 (95% CI 0.87 - 3.68), 0.111
A*25 n (%)	2 (2.27)	2 (1.16)	1.98 (95% CI 0.27 - 14.27), 0.606
A*26 n (%)	3 (3.41)	11 (6.4)	0.52 (95% CI 0.14 - 1.9), 0.394
A*29 n (%)	0 (0)	1 (0.58)	0 (95% CI 0 - NC), 1
A*30 n (%)	7 (7.95)	3 (1.74)	4.87 (95% CI 1.23 - 19.32), 0.034
A*31 n (%)	1 (1.14)	6 (3.49)	0.32 (95% CI 0.04 - 2.68), 0.429
A*32 n (%)	2 (2.27)	11 (6.4)	0.34 (95% CI 0.07 - 1.57), 0.229
A*33 n (%)	1 (1.14)	0 (0)	Inf (95% CI NC - Inf), 0.338
A*66 n (%)	0 (0)	1 (0.58)	0 (95% CI 0 - NC), 1
A*68 n (%)	2 (2.27)	3 (1.74)	1.31 (95% CI 0.21 - 7.99), 1
A*69 n (%)	1 (1.14)	2 (1.16)	0.98 (95% CI 0.09 - 10.93), 1
A*74 n (%)	0 (0)	1 (0.58)	0 (95% CI 0 - NC), 1

n for groups, number of alleles; HLA, human leukocyte antigen; OR, odds ratio; CI, confidence interval; NC, cannot be computed.

Supplementary Table S7. Univariate logistic regression analysis predicting the odds of death based on HLA-B alleles

Deceased:	Yes (n=88)	No (n=172)	OR (95% CI), P-value
B*07 n (%)	4 (4.55)	14 (8.14)	0.54 (95% CI 0.17 - 1.68), 0.28
B*08 n (%)	13 (14.77)	13 (7.56)	2.12 (95% CI 0.94 - 4.8), 0.067
B*13 n (%)	2 (2.27)	6 (3.49)	0.64 (95% CI 0.13 - 3.26), 0.721
B*14 n (%)	3 (3.41)	1 (0.58)	6.04 (95% CI 0.62 - 58.89), 0.114
B*15 n (%)	4 (4.55)	9 (5.23)	0.86 (95% CI 0.26 - 2.88), 1
B*18 n (%)	16 (18.18)	12 (6.98)	2.96 (95% CI 1.33 - 6.58), 0.006
B*27 n (%)	7 (7.95)	15 (8.72)	0.9 (95% CI 0.35 - 2.31), 0.834
B*35 n (%)	9 (10.23)	21 (12.21)	0.82 (95% CI 0.36 - 1.87), 0.636

B*37 n (%)	0 (0)	1 (0.58)	0 (95% CI 0 - NC), 1
B*38 n (%)	3 (3.41)	9 (5.23)	0.64 (95% CI 0.17 - 2.42), 0.756
B*39 n (%)	3 (3.41)	3 (1.74)	1.99 (95% CI 0.39 - 10.06), 0.41
B*40 n (%)	3 (3.41)	7 (4.07)	0.83 (95% CI 0.21 - 3.3), 1
B*41 n (%)	0 (0)	1 (0.58)	0 (95% CI 0 - NC), 1
B*44 n (%)	6 (6.82)	16 (9.3)	0.71 (95% CI 0.27 - 1.89), 0.496
B*47 n (%)	0 (0)	1 (0.58)	0 (95% CI 0 - NC), 1
B*49 n (%)	2 (2.27)	2 (1.16)	1.98 (95% CI 0.27 - 14.27), 0.606
B*50 n (%)	2 (2.27)	8 (4.65)	0.48 (95% CI 0.1 - 2.29), 0.502
B*51 n (%)	4 (4.55)	13 (7.56)	0.58 (95% CI 0.18 - 1.84), 0.352
B*52 n (%)	3 (3.41)	9 (5.23)	0.64 (95% CI 0.17 - 2.42), 0.756
B*53 n (%)	0 (0)	1 (0.58)	0 (95% CI 0 - NC), 1
B*55 n (%)	1 (1.14)	4 (2.33)	0.48 (95% CI 0.05 - 4.39), 0.665
B*56 n (%)	1 (1.14)	1 (0.58)	1.97 (95% CI 0.12 - 31.8), 1
B*57 n (%)	2 (2.27)	5 (2.91)	0.78 (95% CI 0.15 - 4.09), 1
B*58 n (%)	0 (0)	0 (0)	NC (95% CI NC - NC), 1

n for groups, number of alleles; HLA, human leukocyte antigen; OR, odds ratio; CI, confidence interval; NC, cannot be computed.

Supplementary Table S8. Univariate logistic regression analysis predicting the odds of death based on HLA-C alleles

**Deceased:**	**Yes (n=88)**	**No (n=172)**	**OR (95% CI), P-value**
C*01 n (%)	5 (5.68)	7 (4.07)	1.42 (95% CI 0.44 - 4.61), 0.547
C*02 n (%)	7 (7.95)	14 (8.14)	0.98 (95% CI 0.38 - 2.51), 0.959
C*03 n (%)	6 (6.82)	14 (8.14)	0.83 (95% CI 0.31 - 2.23), 0.705
C*04 n (%)	10 (11.36)	32 (18.6)	0.56 (95% CI 0.26 - 1.2), 0.133
C*05 n (%)	2 (2.27)	4 (2.33)	0.98 (95% CI 0.18 - 5.44), 1
C*06 n (%)	6 (6.82)	18 (10.47)	0.63 (95% CI 0.24 - 1.64), 0.336
C*07 n (%)	33 (37.5)	39 (22.67)	2.05 (95% CI 1.17 - 3.58), 0.011
C*08 n (%)	3 (3.41)	1 (0.58)	6.04 (95% CI 0.62 - 58.89), 0.114
C*12 n (%)	11 (12.5)	28 (16.28)	0.73 (95% CI 0.35 - 1.56), 0.419
C*14 n (%)	0 (0)	3 (1.74)	0 (95% CI 0 - NC), 0.553
C*15 n (%)	2 (2.27)	6 (3.49)	0.64 (95% CI 0.13 - 3.26), 0.721
C*16 n (%)	3 (3.41)	6 (3.49)	0.98 (95% CI 0.24 - 4), 1
C*17 n (%)	0 (0)	0 (0)	NC (95% CI NC - NC), 1

n for groups, number of alleles; HLA, human leukocyte antigen; OR, odds ratio; CI, confidence interval; NC, cannot be computed.

Supplementary Table S9. Univariate logistic regression analysis predicting the odds of death based on HLA-DRB1 alleles

Deceased:	Yes (n=88)	No (n=172)	OR (95% CI), P-value
DRB1*01 n (%)	6 (6.82)	22 (12.79)	0.5 (95% CI 0.19 - 1.28), 0.142
DRB1*03 n (%)	16 (18.18)	24 (13.95)	1.37 (95% CI 0.69 - 2.74), 0.371
DRB1*04 n (%)	3 (3.41)	16 (9.3)	0.34 (95% CI 0.1 - 1.21), 0.084
DRB1*07 n (%)	8 (9.09)	25 (14.53)	0.59 (95% CI 0.25 - 1.36), 0.212
DRB1*08 n (%)	2 (2.27)	4 (2.33)	0.98 (95% CI 0.18 - 5.44), 1
DRB1*09 n (%)	0 (0)	1 (0.58)	0 (95% CI 0 - NC), 1
DRB1*10 n (%)	1 (1.14)	2 (1.16)	0.98 (95% CI 0.09 - 10.93), 1
DRB1*11 n (%)	25 (28.41)	30 (17.44)	1.88 (95% CI 1.02 - 3.45), 0.04
DRB1*12 n (%)	0 (0)	1 (0.58)	0 (95% CI 0 - NC), 1
DRB1*13 n (%)	10 (11.36)	13 (7.56)	1.57 (95% CI 0.66 - 3.73), 0.307
DRB1*14 n (%)	4 (4.55)	8 (4.65)	0.98 (95% CI 0.29 - 3.34), 1
DRB1*15 n (%)	4 (4.55)	13 (7.56)	0.58 (95% CI 0.18 - 1.84), 0.352
DRB1*16 n (%)	9 (10.23)	13 (7.56)	1.39 (95% CI 0.57 - 3.4), 0.464

n for groups, number of alleles; HLA, human leukocyte antigen; OR, odds ratio; CI, confidence interval; NC, cannot be computed.

Supplementary Table S10. Univariate logistic regression analysis predicting the odds of death based on HLA-DQB1 alleles

Deceased:	Yes (n=88)	No (n=172)	OR (95% CI), P-value
DQB1*02 n (%)	22 (25)	46 (26.74)	0.91 (95% CI 0.51 - 1.65), 0.762
DQB1*03 n (%)	31 (35.23)	53 (30.81)	1.22 (95% CI 0.71 - 2.1), 0.471
DQB1*04 n (%)	2 (2.27)	3 (1.74)	1.31 (95% CI 0.21 - 7.99), 1
DQB1*05 n (%)	22 (25)	48 (27.91)	0.86 (95% CI 0.48 - 1.55), 0.617
DQB1*06 n (%)	11 (12.5)	22 (12.79)	0.97 (95% CI 0.45 - 2.11), 0.947

n for groups, number of alleles; HLA, human leukocyte antigen; OR, odds ratio; CI, confidence interval; NC, cannot be computed.

Comparison concerning diseased or ICU status

Supplementary Table S11. Univariate logistic regression analysis predicting the odds of death or ICU stay based on HLA-A alleles

Deceased/ICU:	Yes (n=146)	No (n=114)	OR (95% CI), P-value
A*01 n (%)	26 (17.81)	15 (13.16)	1.43 (95% CI 0.72 - 2.85), 0.307

A*02 n (%)	46 (31.51)	31 (27.19)	1.23 (95% CI 0.72 - 2.11), 0.45
A*03 n (%)	10 (6.85)	17 (14.91)	0.42 (95% CI 0.18 - 0.96), 0.034
A*11 n (%)	11 (7.53)	6 (5.26)	1.47 (95% CI 0.53 - 4.09), 0.462
A*23 n (%)	0 (0)	3 (2.63)	0 (95% CI 0 - NC), 0.083
A*24 n (%)	21 (14.38)	14 (12.28)	1.2 (95% CI 0.58 - 2.48), 0.622
A*25 n (%)	2 (1.37)	2 (1.75)	0.78 (95% CI 0.11 - 5.61), 1
A*26 n (%)	7 (4.79)	7 (6.14)	0.77 (95% CI 0.26 - 2.26), 0.633
A*29 n (%)	1 (0.68)	0 (0)	Inf (95% CI NC - Inf), 1
A*30 n (%)	7 (4.79)	3 (2.63)	1.86 (95% CI 0.47 - 7.37), 0.52
A*31 n (%)	2 (1.37)	5 (4.39)	0.3 (95% CI 0.06 - 1.59), 0.246
A*32 n (%)	5 (3.42)	8 (7.02)	0.47 (95% CI 0.15 - 1.48), 0.187
A*33 n (%)	1 (0.68)	0 (0)	Inf (95% CI NC - Inf), 1
A*66 n (%)	0 (0)	1 (0.88)	0 (95% CI 0 - NC), 0.438
A*68 n (%)	3 (2.05)	2 (1.75)	1.17 (95% CI 0.19 - 7.15), 1
A*69 n (%)	3 (2.05)	0 (0)	Inf (95% CI NC - Inf), 0.259
A*74 n (%)	1 (0.68)	0 (0)	Inf (95% CI NC - Inf), 1

n for groups, number of alleles; HLA, human leukocyte antigen; ICU, intensive care unit; OR, odds ratio; CI, confidence interval; NC, cannot be computed.

Supplementary Table S12. Univariate logistic regression analysis predicting the odds of death or ICU stay based on HLA-B alleles

Deceased/ICU:	Yes (n=146)	No (n=114)	OR (95% CI), P-value
B*07 n (%)	8 (5.48)	10 (8.77)	0.6 (95% CI 0.23 - 1.58), 0.299
B*08 n (%)	17 (11.64)	9 (7.89)	1.54 (95% CI 0.66 - 3.59), 0.317
B*13 n (%)	3 (2.05)	5 (4.39)	0.46 (95% CI 0.11 - 1.96), 0.304
B*14 n (%)	4 (2.74)	0 (0)	Inf (95% CI NC - Inf), 0.133
B*15 n (%)	9 (6.16)	4 (3.51)	1.81 (95% CI 0.54 - 6.02), 0.33
B*18 n (%)	20 (13.7)	8 (7.02)	2.1 (95% CI 0.89 - 4.97), 0.085
B*27 n (%)	11 (7.53)	11 (9.65)	0.76 (95% CI 0.32 - 1.83), 0.543
B*35 n (%)	13 (8.9)	17 (14.91)	0.56 (95% CI 0.26 - 1.2), 0.132
B*37 n (%)	0 (0)	1 (0.88)	0 (95% CI 0 - NC), 0.438
B*38 n (%)	6 (4.11)	6 (5.26)	0.77 (95% CI 0.24 - 2.46), 0.66
B*39 n (%)	3 (2.05)	3 (2.63)	0.78 (95% CI 0.15 - 3.92), 1
B*40 n (%)	7 (4.79)	3 (2.63)	1.86 (95% CI 0.47 - 7.37), 0.52
B*41 n (%)	1 (0.68)	0 (0)	Inf (95% CI NC - Inf), 1
B*44 n (%)	10 (6.85)	12 (10.53)	0.62 (95% CI 0.26 - 1.5), 0.29
B*47 n (%)	1 (0.68)	0 (0)	Inf (95% CI NC - Inf), 1
B*49 n (%)	2 (1.37)	2 (1.75)	0.78 (95% CI 0.11 - 5.61), 1
B*50 n (%)	3 (2.05)	7 (6.14)	0.32 (95% CI 0.08 - 1.27), 0.11

B*51 n (%)	9 (6.16)	8 (7.02)	0.87 (95% CI 0.32 - 2.33), 0.782
B*52 n (%)	9 (6.16)	3 (2.63)	2.43 (95% CI 0.64 - 9.19), 0.178
B*53 n (%)	0 (0)	1 (0.88)	0 (95% CI 0 - NC), 0.438
B*55 n (%)	4 (2.74)	1 (0.88)	3.18 (95% CI 0.35 - 28.88), 0.389
B*56 n (%)	1 (0.68)	1 (0.88)	0.78 (95% CI 0.05 - 12.6), 1
B*57 n (%)	5 (3.42)	2 (1.75)	1.99 (95% CI 0.38 - 10.43), 0.472
B*58 n (%)	0 (0)	0 (0)	NC (95% CI NC - NC), 1

n for groups, number of alleles; HLA, human leukocyte antigen; ICU, intensive care unit; OR, odds ratio; CI, confidence interval; NC, cannot be computed.

Supplementary Table S13. Univariate logistic regression analysis predicting the odds of death or ICU stay based on HLA-C alleles

Deceased/ICU:	Yes (n=146)	No (n=114)	OR (95% CI), P-value
C*01 n (%)	7 (4.79)	5 (4.39)	1.1 (95% CI 0.34 - 3.55), 0.876
C*02 n (%)	10 (6.85)	11 (9.65)	0.69 (95% CI 0.28 - 1.68), 0.411
C*03 n (%)	14 (9.59)	6 (5.26)	1.91 (95% CI 0.71 - 5.14), 0.194
C*04 n (%)	16 (10.96)	26 (22.81)	0.42 (95% CI 0.21 - 0.82), 0.01
C*05 n (%)	3 (2.05)	3 (2.63)	0.78 (95% CI 0.15 - 3.92), 1
C*06 n (%)	11 (7.53)	13 (11.4)	0.63 (95% CI 0.27 - 1.47), 0.285
C*07 n (%)	46 (31.51)	26 (22.81)	1.56 (95% CI 0.89 - 2.72), 0.12
C*08 n (%)	4 (2.74)	0 (0)	Inf (95% CI NC - Inf), 0.133
C*12 n (%)	23 (15.75)	16 (14.04)	1.15 (95% CI 0.57 - 2.29), 0.7
C*14 n (%)	2 (1.37)	1 (0.88)	1.57 (95% CI 0.14 - 17.53), 1
C*15 n (%)	4 (2.74)	4 (3.51)	0.77 (95% CI 0.19 - 3.17), 0.733
C*16 n (%)	6 (4.11)	3 (2.63)	1.59 (95% CI 0.39 - 6.48), 0.735
C*17 n (%)	0 (0)	0 (0)	NC (95% CI NC - NC), 1

n for groups, number of alleles; HLA, human leukocyte antigen; ICU, intensive care unit; OR, odds ratio; CI, confidence interval; NC, cannot be computed.

Supplementary Table S14. Univariate logistic regression analysis predicting the odds of death or ICU stay based on HLA-DRB1 alleles

Deceased/ICU:	Yes (n=146)	No (n=114)	OR (95% CI), P-value
DRB1*01 n (%)	15 (10.27)	13 (11.4)	0.89 (95% CI 0.41 - 1.95), 0.771
DRB1*03 n (%)	23 (15.75)	17 (14.91)	1.07 (95% CI 0.54 - 2.11), 0.852
DRB1*04 n (%)	7 (4.79)	12 (10.53)	0.43 (95% CI 0.16 - 1.13), 0.078
DRB1*07 n (%)	14 (9.59)	19 (16.67)	0.53 (95% CI 0.25 - 1.11), 0.089
DRB1*08 n (%)	4 (2.74)	2 (1.75)	1.58 (95% CI 0.28 - 8.77), 0.698
DRB1*09 n (%)	0 (0)	1 (0.88)	0 (95% CI 0 - NC), 0.438

DRB1*10 n (%)	1 (0.68)	2 (1.75)	0.39 (95% CI 0.03 - 4.31), 0.583
DRB1*11 n (%)	32 (21.92)	23 (20.18)	1.11 (95% CI 0.61 - 2.03), 0.733
DRB1*12 n (%)	1 (0.68)	0 (0)	Inf (95% CI NC - Inf), 1
DRB1*13 n (%)	17 (11.64)	6 (5.26)	2.37 (95% CI 0.9 - 6.23), 0.072
DRB1*14 n (%)	8 (5.48)	4 (3.51)	1.59 (95% CI 0.47 - 5.43), 0.452
DRB1*15 n (%)	12 (8.22)	5 (4.39)	1.95 (95% CI 0.67 - 5.71), 0.215
DRB1*16 n (%)	12 (8.22)	10 (8.77)	0.93 (95% CI 0.39 - 2.24), 0.874

n for groups, number of alleles; HLA, human leukocyte antigen; ICU, intensive care unit; OR, odds ratio; CI, confidence interval; NC, cannot be computed.

Supplementary Table S15. Univariate logistic regression analysis predicting the odds of death or ICU stay based on HLA-DQB1 alleles

Deceased/ICU:	Yes (n=146)	No (n=114)	OR (95% CI), P-value
DQB1*02 n (%)	35 (23.97)	33 (28.95)	0.77 (95% CI 0.44 - 1.35), 0.365
DQB1*03 n (%)	43 (29.45)	41 (35.96)	0.74 (95% CI 0.44 - 1.25), 0.265
DQB1*04 n (%)	4 (2.74)	1 (0.88)	3.18 (95% CI 0.35 - 28.88), 0.389
DQB1*05 n (%)	40 (27.4)	30 (26.32)	1.06 (95% CI 0.61 - 1.84), 0.845
DQB1*06 n (%)	24 (16.44)	9 (7.89)	2.3 (95% CI 1.02 - 5.16), 0.04

n for groups, number of alleles; HLA, human leukocyte antigen; ICU, intensive care unit; OR, odds ratio; CI, confidence interval; NC, cannot be computed.

Comparisons concerning oxygen therapy

Supplementary Table S16. Univariate logistic regression analysis predicting the odds of oxygen therapy based on HLA-A alleles

With oxygen:	Yes (n=144)	No (n=92)	OR (95% CI), P-value
A*01 n (%)	26 (18.06)	12 (13.04)	1.47 (95% CI 0.7 - 3.08), 0.307
A*02 n (%)	47 (32.64)	26 (28.26)	1.23 (95% CI 0.69 - 2.18), 0.478
A*03 n (%)	11 (7.64)	15 (16.3)	0.42 (95% CI 0.19 - 0.97), 0.038
A*11 n (%)	7 (4.86)	8 (8.7)	0.54 (95% CI 0.19 - 1.53), 0.239
A*23 n (%)	2 (1.39)	1 (1.09)	1.28 (95% CI 0.11 - 14.34), 1
A*24 n (%)	20 (13.89)	8 (8.7)	1.69 (95% CI 0.71 - 4.02), 0.229
A*25 n (%)	2 (1.39)	2 (2.17)	0.63 (95% CI 0.09 - 4.58), 0.644
A*26 n (%)	11 (7.64)	3 (3.26)	2.45 (95% CI 0.67 - 9.04), 0.165
A*29 n (%)	1 (0.69)	0 (0)	Inf (95% CI NC - Inf), 1
A*30 n (%)	2 (1.39)	3 (3.26)	0.42 (95% CI 0.07 - 2.55), 0.381
A*31 n (%)	3 (2.08)	3 (3.26)	0.63 (95% CI 0.12 - 3.2), 0.68

A*32 n (%)	6 (4.17)	7 (7.61)	0.53 (95% CI 0.17 - 1.62), 0.258
A*33 n (%)	1 (0.69)	0 (0)	Inf (95% CI NC - Inf), 1
A*66 n (%)	0 (0)	1 (1.09)	0 (95% CI 0 - NC), 0.39
A*68 n (%)	2 (1.39)	3 (3.26)	0.42 (95% CI 0.07 - 2.55), 0.381
A*69 n (%)	2 (1.39)	0 (0)	Inf (95% CI NC - Inf), 0.522

n for groups, number of alleles; HLA, human leukocyte antigen; OR, odds ratio; CI, confidence interval; NC, cannot be computed.

Supplementary Table S17. Univariate logistic regression analysis predicting the odds of oxygen therapy based on HLA-B alleles

**With oxygen:**	**Yes (n=144)**	**No (n=92)**	**OR (95% CI), P-value**
B*07 n (%)	10 (6.94)	7 (7.61)	0.91 (95% CI 0.33 - 2.47), 0.847
B*08 n (%)	11 (7.64)	11 (11.96)	0.61 (95% CI 0.25 - 1.47), 0.266
B*13 n (%)	2 (1.39)	5 (5.43)	0.25 (95% CI 0.05 - 1.29), 0.113
B*14 n (%)	2 (1.39)	0 (0)	Inf (95% CI NC - Inf), 0.522
B*15 n (%)	8 (5.56)	4 (4.35)	1.29 (95% CI 0.38 - 4.43), 0.77
B*18 n (%)	15 (10.42)	8 (8.7)	1.22 (95% CI 0.5 - 3.01), 0.664
B*27 n (%)	13 (9.03)	9 (9.78)	0.92 (95% CI 0.37 - 2.24), 0.846
B*35 n (%)	16 (11.11)	11 (11.96)	0.92 (95% CI 0.41 - 2.08), 0.842
B*37 n (%)	0 (0)	1 (1.09)	0 (95% CI 0 - NC), 0.39
B*38 n (%)	9 (6.25)	3 (3.26)	1.98 (95% CI 0.52 - 7.51), 0.376
B*39 n (%)	3 (2.08)	2 (2.17)	0.96 (95% CI 0.16 - 5.84), 1
B*40 n (%)	7 (4.86)	2 (2.17)	2.3 (95% CI 0.47 - 11.32), 0.488
B*41 n (%)	1 (0.69)	0 (0)	Inf (95% CI NC - Inf), 1
B*44 n (%)	11 (7.64)	9 (9.78)	0.76 (95% CI 0.3 - 1.92), 0.564
B*47 n (%)	1 (0.69)	0 (0)	Inf (95% CI NC - Inf), 1
B*49 n (%)	2 (1.39)	2 (2.17)	0.63 (95% CI 0.09 - 4.58), 0.644
B*50 n (%)	5 (3.47)	5 (5.43)	0.63 (95% CI 0.18 - 2.22), 0.518
B*51 n (%)	10 (6.94)	5 (5.43)	1.3 (95% CI 0.43 - 3.93), 0.643
B*52 n (%)	8 (5.56)	4 (4.35)	1.29 (95% CI 0.38 - 4.43), 0.77
B*53 n (%)	1 (0.69)	0 (0)	Inf (95% CI NC - Inf), 1
B*55 n (%)	3 (2.08)	1 (1.09)	1.94 (95% CI 0.2 - 18.9), 1
B*56 n (%)	1 (0.69)	1 (1.09)	0.64 (95% CI 0.04 - 10.3), 1
B*57 n (%)	5 (3.47)	2 (2.17)	1.62 (95% CI 0.31 - 8.52), 0.708
B*58 n (%)	0 (0)	0 (0)	NC (95% CI NC - NC), 1

n for groups, number of alleles; HLA, human leukocyte antigen; OR, odds ratio; CI, confidence interval; NC, cannot be computed.

Supplementary Table S18. Univariate logistic regression analysis predicting the odds of oxygen therapy based on HLA-C alleles

With oxygen:	Yes (n=144)	No (n=92)	OR (95% CI), P-value
C*01 n (%)	8 (5.56)	4 (4.35)	1.29 (95% CI 0.38 - 4.43), 0.77
C*02 n (%)	11 (7.64)	10 (10.87)	0.68 (95% CI 0.28 - 1.67), 0.395
C*03 n (%)	11 (7.64)	7 (7.61)	1 (95% CI 0.37 - 2.69), 0.993
C*04 n (%)	21 (14.58)	17 (18.48)	0.75 (95% CI 0.37 - 1.52), 0.427
C*05 n (%)	2 (1.39)	3 (3.26)	0.42 (95% CI 0.07 - 2.55), 0.381
C*06 n (%)	11 (7.64)	12 (13.04)	0.55 (95% CI 0.23 - 1.31), 0.172
C*07 n (%)	39 (27.08)	23 (25)	1.11 (95% CI 0.61 - 2.03), 0.723
C*08 n (%)	2 (1.39)	0 (0)	Inf (95% CI NC - Inf), 0.522
C*12 n (%)	26 (18.06)	13 (14.13)	1.34 (95% CI 0.65 - 2.76), 0.428
C*14 n (%)	3 (2.08)	0 (0)	Inf (95% CI NC - Inf), 0.283
C*15 n (%)	5 (3.47)	1 (1.09)	3.27 (95% CI 0.38 - 28.48), 0.409
C*16 n (%)	5 (3.47)	2 (2.17)	1.62 (95% CI 0.31 - 8.52), 0.708
C*17 n (%)	0 (0)	0 (0)	NC (95% CI NC - NC), 1

n for groups, number of alleles; HLA, human leukocyte antigen; OR, odds ratio; CI, confidence interval; NC, cannot be computed.

Supplementary Table S19. Univariate logistic regression analysis predicting the odds of oxygen therapy based on HLA-DRB1 alleles

With oxygen:	Yes (n=144)	No (n=92)	OR (95% CI), P-value
DRB1*01 n (%)	17 (11.81)	9 (9.78)	1.23 (95% CI 0.53 - 2.9), 0.628
DRB1*03 n (%)	17 (11.81)	18 (19.57)	0.55 (95% CI 0.27 - 1.13), 0.102
DRB1*04 n (%)	11 (7.64)	7 (7.61)	1 (95% CI 0.37 - 2.69), 0.993
DRB1*07 n (%)	14 (9.72)	15 (16.3)	0.55 (95% CI 0.25 - 1.21), 0.133
DRB1*08 n (%)	6 (4.17)	0 (0)	Inf (95% CI NC - Inf), 0.084
DRB1*09 n (%)	0 (0)	1 (1.09)	0 (95% CI 0 - NC), 0.39
DRB1*10 n (%)	2 (1.39)	1 (1.09)	1.28 (95% CI 0.11 - 14.34), 1
DRB1*11 n (%)	30 (20.83)	18 (19.57)	1.08 (95% CI 0.56 - 2.08), 0.813
DRB1*12 n (%)	1 (0.69)	0 (0)	Inf (95% CI NC - Inf), 1
DRB1*13 n (%)	16 (11.11)	5 (5.43)	2.17 (95% CI 0.77 - 6.16), 0.135
DRB1*14 n (%)	8 (5.56)	3 (3.26)	1.75 (95% CI 0.45 - 6.76), 0.535
DRB1*15 n (%)	12 (8.33)	4 (4.35)	2 (95% CI 0.62 - 6.4), 0.235
DRB1*16 n (%)	10 (6.94)	11 (11.96)	0.55 (95% CI 0.22 - 1.35), 0.187

n for groups, number of allele; HLA, human leukocyte antigen; OR, odds ratio; CI, confidence interval; NC, cannot be computed.

Supplementary Table S20. Univariate logistic regression analysis predicting the odds of oxygen therapy based on HLA-DQB1 alleles

With oxygen:	Yes (n=144)	No (n=92)	OR (95% CI), P-value
DQB1*02 n (%)	28 (19.44)	31 (33.7)	0.47 (95% CI 0.26 - 0.86), 0.014
DQB1*03 n (%)	48 (33.33)	28 (30.43)	1.14 (95% CI 0.65 - 2.01), 0.642
DQB1*04 n (%)	5 (3.47)	0 (0)	Inf (95% CI NC - Inf), 0.16
DQB1*05 n (%)	40 (27.78)	25 (27.17)	1.03 (95% CI 0.57 - 1.85), 0.919
DQB1*06 n (%)	23 (15.97)	8 (8.7)	2 (95% CI 0.85 - 4.68), 0.107

n for groups, number of alleles; HLA, human leukocyte antigen; OR, odds ratio; CI, confidence interval; NC, cannot be computed.

Multiple logistic regressions

Supplementary Table S21. Multiple logistic regressions predicting deceased within the severe group, based on HLA allele A*30, adjusted for age, sex, number of comorbidities, and vaccination.

	OR adjusted	(95% CI)	p
Age (years)	1.05	(1.01 - 1.09)	0.026
Sex (M vs. F)	5.46	(2.02 - 16.19)	0.001
Number comorbidities	5.07	(2.84 - 10.15)	< 0.001
Vaccinated (Yes vs. No)	0.08	(0.01 - 0.41)	0.009
A*30 (Yes vs. No)	14.05	(0.59 - 238.42)	0.073

Supplementary Table S22. Multiple logistic regressions predicting deceased within the severe group, based on HLA allele B*18, adjusted for age, sex, number of comorbidities, and vaccination.

	OR adjusted	(95% CI)	p
Age (years)	1.04	(1.01 - 1.09)	0.032
Sex (M vs. F)	5.56	(2.08 - 16.41)	0.001
Number comorbidities	4.84	(2.75 - 9.58)	< 0.001
Vaccinated (Yes vs. No)	0.13	(0.02 - 0.55)	0.014
B*18 (Yes vs. No)	1.46	(0.36 - 5.98)	0.597

Supplementary Table S23. Multiple logistic regressions predicting deceased within the severe group, based on HLA allele C*07, adjusted for age, sex, number of comorbidities, and vaccination.

	OR adjusted	(95% CI)	p
Age (years)	1.04	(1 - 1.08)	0.042
Sex (M vs. F)	5.17	(1.91 - 15.34)	0.002
Number comorbidities	5.09	(2.88 - 10.06)	< 0.001

Vaccinated (Yes vs. No)	0.13	(0.02 - 0.55)	0.013
C*07 (Yes vs. No)	1.54	(0.49 - 4.85)	0.456

Supplementary Table S24. Multiple logistic regressions predicting deceased within the severe group, based on HLA allele DRB1*11, adjusted for age, sex, number of comorbidities, and vaccination.

	OR adjusted	(95% CI)	p
Age (years)	1.04	(1.01 - 1.09)	0.033
Sex (M vs. F)	5.54	(2.08 - 16.3)	< 0.001
Number comorbidities	5.01	(2.83 - 9.86)	< 0.001
Vaccinated (Yes vs. No)	0.13	(0.02 - 0.54)	0.013
DRB1*11 (Yes vs. No)	0.89	(0.21 - 3.56)	0.874

Supplementary Table S25. Multiple logistic regressions predicting deceased, or ICU stay within severe group, based on HLA allele A*03, adjusted for age, sex, and number of comorbidities.

	OR adjusted	(95% CI)	p
Age (years)	1.06	(1.03 - 1.1)	< 0.001
Sex (M vs. F)	3.99	(1.7 - 9.81)	0.002
Number comorbidities	5.83	(3.2 - 11.75)	< 0.001
Vaccinated (Yes vs. No)	0.32	(0.12 - 0.81)	0.019
A*03 (Yes vs. No)	0.14	(0.02 - 0.77)	0.036

Supplementary Table S26. Multiple logistic regressions predicting deceased, or ICU stay within severe group, based on HLA allele C*04, adjusted for age, sex, and number of comorbidities.

	OR adjusted	(95% CI)	p
Age (years)	1.06	(1.02 - 1.1)	< 0.001
Sex (M vs. F)	4.41	(1.9 - 10.79)	< 0.001
Number comorbidities	5.57	(3.08 - 11.12)	< 0.001
Vaccinated (Yes vs. No)	0.36	(0.14 - 0.9)	0.032
C*04 (Yes vs. No)	0.48	(0.15 - 1.5)	0.218

Supplementary Table S27. Multiple logistic regressions predicting deceased, or ICU stay within severe group, based on HLA allele DQB1*06, adjusted for age, sex, and number of comorbidities.

	OR adjusted	(95% CI)	p
Age (years)	1.06	(1.03 - 1.1)	< 0.001
Sex (M vs. F)	4.27	(1.83 - 10.46)	0.001
Number comorbidities	5.59	(3.1 - 11.08)	< 0.001

Vaccinated (Yes vs. No)	0.34	(0.13 - 0.87)	0.027
DQB1*06 (Yes vs. No)	3.2	(0.95 - 11.46)	0.065

Supplementary Table S28. Multiple logistic regressions predicting oxygen therapy within the severe group, based on HLA allele A*03, adjusted for age, sex, number of comorbidities, and vaccination.

	OR adjusted	(95% CI)	p
Age (years)	1.09	(1.05 - 1.12)	< 0.001
Sex (M vs. F)	1.24	(0.59 - 2.61)	0.565
Number comorbidities	1.05	(0.7 - 1.61)	0.803
Vaccinated (Yes vs. No)	0.22	(0.1 - 0.49)	< 0.001
A*03 (Yes vs. No)	0.26	(0.07 - 0.85)	0.032

Supplementary Table S29. Multiple logistic regressions predicting oxygen therapy within the severe group, based on HLA allele DQB1*02, adjusted for age, sex, number of comorbidities, and vaccination.

	OR adjusted	(95% CI)	p
Age (years)	1.09	(1.06 - 1.13)	< 0.001
Sex (M vs. F)	1.16	(0.55 - 2.45)	0.693
Number comorbidities	1.05	(0.69 - 1.6)	0.835
Vaccinated (Yes vs. No)	0.25	(0.11 - 0.54)	< 0.001
DQB1*02 (Yes vs. No)	0.31	(0.13 - 0.7)	0.006
